# Development of global visual processing: From the retina to the perceptive field

**DOI:** 10.1371/journal.pone.0238246

**Published:** 2020-08-31

**Authors:** Ravid Doron, Maria Lev, Tamara Wygnanski-Jaffe, Iris Moroz, Uri Polat

**Affiliations:** 1 Department of Optometry and Vision Science, Hadassah Academic College, Jerusalem, Israel; 2 The School of Optometry and Vision Science, The Mina & Everard Goodman Faculty of Life Sciences, Bar Ilan University, Ramat-Gan, Israel; 3 Department of Ophthalmology, Sheba Medical Center, Tel Hashomer, Israel; 4 Sackler Faculty of Medicine, Tel Aviv University, Tel Aviv, Israel; 5 Department of Eye Imaging, Goldschleger Eye Research Institute, Sheba Medical Center, Tel Hashomer, Israel; University Hospitals Cleveland, UNITED STATES

## Abstract

Young children exhibit poorer visual performance than adults due to immaturity of the fovea and of the fundamental processing of visual functions such as masking and crowding. Recent studies suggest that masking and crowding are closely related to the size of the fundamental processing unit—the perceptive field (PF). However, while it is known that the retina and basic visual functions develop throughout childhood, it is not clear whether and how changes in the size of the PF affect masking and crowding. Furthermore, no retinal and perceptual development data have been collected from the same cohort and time. Here we explored the developmental process of the PF and the basic visual functions. Psychophysical and imaging methods were used to test visual functions and foveal changes in participants ranging from 3–17 years old. Lateral masking, crowding and contrast sensitivity were tested using computerized tasks. Foveal measurements were obtained from spectral-domain optical coherence tomography (OCT). The children patterns below 6 years exhibited high crowding, while the expected facilitation was found only at a larger target-flanker distance than required for children above 6 years, who exhibited the typical adult. Foveal thickness and macular volume for the children below 6 years were significantly lower than for the older group. Significant correlation was found for contrast sensitivity, foveal thickness and macular volume with age and between contrast sensitivity and foveal thickness. Our data suggest that the developmental processes at the retina and visual cortex occur in the same age range. Thus, in parallel to maturation of the PF, which enables reduction in crowding, foveal development contributes to increasing contrast sensitivity.

## Introduction

The visual system undergoes a sequence of development of various visual functions, each maturing at a different age [1–3]. Visual performance depends on the critical period [4] and is linked to the anatomical development of the visual system [5]. It is generally accepted that both the human fovea and visual cortex mature in synchrony to enable visual perception [6, 7].

Foveal development was thought to be completed between 11 months and 5 years of age [8–11], but it was recently demonstrated that this development continues into adulthood [12]. Normal retinal maturation involves displacement of the retinal layers in three developmental steps [8, 13]; peripheral migration or displacement of the inner retinal layers that form the foveal depression, central-ward migration of cones and their elongation, and diminution in the thickness of the cones, which increases the density of foveolar cell packing. Foveola width and cone diameter reach the adult level at around 45 months of age, but outer segment length and cone packing density continue to develop afterwards [8, 13]. This process reflects the maturation of the retina and accordingly affects visual acuity and contrast sensitivity [14–16].

In the visual cortex, synaptic density increases from birth reaching adult-like levels by the age of 4 years, varying according to cortical region [17]. The primary visual structural networks appear by early childhood but undergo significant expansion in early adolescence before a contraction in late adolescence [18].

The processing of visual information from the retina to the primary visual cortex is based on a feedforward network, in which receptive fields) RFs), which integrate input from localized parts of the visual field, feed the information to the next processing level, enabling encoding of global visual perception [19]. It was suggested that in the bottom-up information processing RFs not only enable the simple feed-forward processes (i.e., lines, spots or bars), but are also involved in complex processes that predict coding of complex scenes [20].

Though RF responses already exist at birth, normal maturation requires appropriate visual stimuli within a sensitive period [21]. RF processing is affected by feedforward stimuli, by lateral interactions (excitatory and inhibitory) that allow integration of visual information through long-range horizontal connections [22–26] and also by feedback processing [27]. Anatomical studies of human cortex have found that the horizontal connections exist at birth and develop progressively for up to 15 months afterwards [28]. Synapse production reaches adult values during late childhood and early adolescence [17, 29].

Excitatory and inhibitory interactions between neighboring cortical neurons (lateral interactions [30, 31]) can create facilitation or suppression and can enhance human perceptual ability [30]. Lateral masking is a perceptual task showing that the visibility of a local target (Gabor patch, GP) can be modified when it is presented between two collinear masks (global percepts) [30–32]. Facilitation is found when the target-flanker is positioned at distance considered to be greater than the RF, while suppression is found for smaller distances [33]. The suppression effect, demonstrated with the lateral masking paradigm, may also be involved in the crowding effect [30] (when objects with similar properties are very close to one another, it is difficult to identify them [34]).

A receptive field is the region of the visual field in which a stimulus modulates the activity of a single neuron; its extent is measured physiologically (spike count) [35, 36]. The perceptive field (PF) refers to the region of the visual field in which a stimulus evokes neural activity (not confined to a single neuron) and is measured psychophysically (perceptual response) [36]. Psychophysical reverse correlation suggested that visual PF (measured using Gabor signals) is the psychophysical analog to the visual RF (cortical simple cell) [37, 38]. Furthermore, lateral masking studies using Gabor patches provide an estimation of PF size [30, 39] that matched RF size [40, 41].

Recent psychophysical studies in adults show that the suppressive zone in a lateral masking tasks is indicative of PF size [37]) and that this size increases with retinal eccentricity (larger in the periphery). Therefore, facilitation at the periphery arises from target-flanker distances larger than those at the fovea (more than 3 wavelengths (λ)) [33, 42]. It was also suggested that facilitation at larger target-flanker distances than 3 λ may characterize the young central retina, where the foveal PF is possibly not yet mature [3] and its contrast sensitivity is tuned to low spatial frequencies [43].

As mentioned above, visual maturation depends on retinal and visual cortex development [44, 45]. Our recent study on young children [3] suggested mutual neuronal mechanisms for some visual functions that showed a sequential development over the maturation period. Cortical processing is influenced by a shift from a high level of crowding, high contour detection threshold, and lateral suppression towards the adult level of crowding and contour integration, reaching it by age 5–6 years. In parallel, there is an increase in contrast sensitivity that is influenced by foveal maturation. Our results suggested that the high suppression at a young age may be effected by a larger suppression zone that gradually decreases with age, revealing the facilitatory effect. Thus, the lack of facilitation in young children is due to PFs size being larger than 3λ (increased suppression zone), resulting in suppression only.

Doron et al. (2015) suggested that the absence of facilitation at the normal target-flanker distance in young children is due to a larger PF, but a possible effect of facilitation may be found at larger target-flanker separations. In other words, children aged 6 may exhibit facilitation at 4λ, whereas facilitation at 3λ appears around 6.5 years. This idea was based on the suggestion that the fovea of children and strabismic amblyopies is still immature, resembling the periphery of normal adults [46]. Furthermore, some studies in infants have demonstrated the presence of only suppressive interactions with no facilitation effect [47, 48]. A study on infant macaques reported no difference in the suppression amplitude over the course of the development and suggested that facilitation develops slowly over the first year after birth due to a top-down process [49]. However, the suggestion by Doron et al. (2015) of a link between PF size and perceptual functions called for further research. In addition, although the literature describes some changes in the visual cortex (perception) and the retina during development, these changes (i.e., contrast sensitivity vs. foveal development and crowding / masking vs PF size) have not been examined in one cohort at one time in order to better understand the maturation process affecting visual functions.

We thus examined the crowding effect and lateral masking in children with normal vision using varied target-flanker separations to reveal the suppression zone and the size of the PF. In addition we measured foveal parameters (thickness, width and volume) in the same cohort using spectral-domain optical coherence tomography (SD-OCT) [50] to test changes occurring over the time known to be linked to a decrease in contrast sensitivity.

## Methods

### Subjects

Healthy children 3 to 17 years of age were recruited for this study. According to our recent study that showed maturation of visual perceptual occurs at around 6 years of age, i.e., significant differences in lateral masking between young children and teenagers [3], we defined two groups of participants: age 3–6 years (6 years and 11 months) was defined as young, 7–17 was defined as teenagers. The Inclusion–exclusion criteria for this study were normal or corrected-to-normal visual acuity and stereo acuity relative to age, no ocular pathology, eye surgery or medication that could affect vision.

Prior to the experimental procedure, all subjects were tested by an optometrist and were asked about their ocular and medical history for assessing by the criteria for participating in the study. Additionally, to ensure that young children did not show latent refractive error they were examined by an ophthalmologist with cycloplegic refraction. Two standard clinical tests determined whether the subject was eligible to participate: 1) Distance visual acuity (VA) was measured according to its best visual correction at a distance of 3 m, using a modified *Bailey–Lovie (LogMAR) chart* (ETDRS) for the teenagers or LEA / Landolt C charts for the young children. To avoid crowding, visual acuity was measured using a "window" (paper aperture) allowing exposure of a single letter at a time. 2) *A Randot Stereo test* was also performed to rule out amblyopia and strabismus and to verify binocular vision.

This study was conducted in compliance with the Helsinki Declaration and was approved by the Human Research Committee at the Sheba Medical Center. Written informed consent was obtained from all parents after they were informed about the nature of the procedures.

### Apparatus

Psychophysical stimuli were presented on a Philips 107P color monitor using a PC (1024 X 768 pixels at a 100 Hz refresh rate; gamma correction was applied). The effective size of the monitor screen was 32 X 23 cm, which, at a viewing distance of 150 cm, subtended a visual angle of 9 X 12°. The experiments were conducted in a dark environment, in which the only ambient light came from the monitor (except for the visual acuity test). The OCT scans were made using a cirrus SD-OCT Carl Zeiss Meditec, software version 6.0.2.01 in a dark room.

### Experimental procedures

We tested the crowding effect using the “Tumbling-E patterns (TeVA) test” paradigm [51]. The stimulus were black E-patterns on a white background, corresponding to a subset of the LogMar chart. The baseline (TeVA = 0) pattern size corresponded to the baseline of the LogMar chart (i.e., 6/6 vision). Viewing distance was 300 cm. Children were asked to detect the direction of the central E (up, down, right and left). A staircase in which each step was modified by 0.1 log unit was used to determine the size for 50% correct level (chance level was 25%). The performance when the central E was presented alone (TeVA single) was measured separately. Crowding was calculated as TeVA elevation = crowded––single (difference on a log scale), i.e., normalizing the crowded condition by the acuity of a single letter.

The lateral masking task was used to test elevation of contrast threshold (contrast detection of a single target in the presence of a mask, with targets normalized to the target condition, i.e., lateral interaction) and contrast sensitivity (contrast detection of a single target) [30]. This task used vertically oriented Gabor patches (GPs) consisting of a low contrast target and two high contrast collinear flankers (masks) at 5 target-flanker distances (2, 3, 4, 4.5 and 5 wavelengths (λ)) (See S2 Fig). Spatial frequency was 9 cpd, specially chosen to lie within the range of contrast sensitivity that both the younger and the older participants could discern [3, 30, 31, 43, 52, 53]. Viewing distance was 150 cm.

Responses consisted of two alternative spatial forced choices with the keyboard's arrows. A response was required in each trial; subjects were asked to detect the target displayed on the right or left side of the screen. Contrast thresholds were measured with a staircase method that converged to 79% correct [54] and increased by 0.1 log units (26%) after an erroneous response and decreased by the same amount after three consecutive correct responses. Approximately 40 trials were required to estimate the threshold in each block. Threshold elevation was determined by measuring the contrast detection of a localized target in the presence of a mask normalized to the target alone. Orthogonal flankers were positioned at a distance of 15λ above and below the target to minimize spatial and temporal uncertainty [30, 33, 42, 55, 56]. The tests were adapted for children with static presentation of the stimuli to neutralize attentional bias as much as possible. Negative feedback (audio) was given for incorrect answers, but positive rewards (sticker or sweet snack) were given at the end or sometimes during the experiment When necessary (particularly with very young children), to avoid finger mistakes, the researcher (the first author) sat with his back to the monitor, thus avoiding bias, and pressed the keyboard according to the child's answers. Viewing was direct and binocular.

To measure structural parameters of the fovea a subgroup of the participants from both ends of the age range were tested using optical coherence tomography (SD-OCT) [50]. When possible, we tested both eyes of each participant. For some children (especially the youngest) we tested only one eye because of the child's impatience. The OCT scans were carried out at the Sheba Medical Center in collaboration with a specialist imaging ophthalmologist [IM]. The scans were performed with no eye dilation. Macular Cube 512 A scans X 128 A scans with a 6mm square grid protocols [57, 58] were made with the highest signal intensity, no concentration errors, and minimal segmentation errors. During the scanning procedure the examiner could observe the fundus and continuously control fixation. Some measurements were taken from HD 5 lines raster 1024 A scans in 6 mm line protocol instead of the Macular Cube map. Foveal thickness was measured from the inner retinal reflex on the inner limiting membrane (ILM) up to the superior border of the RPE line zone and defined as the average thickness in the central 1000μm diameter [59]. Macular volume was defined as the sum of all volumes of all nine sections, i.e., 6 mm square [60]. The parafoveal width was measured at the sides of the highest points of the foveal pit, from the nasal to the temporal retinal zone (See S1 Fig). These measures were examined by the same specialist imaging ophthalmologist, who was kept ignorant of the participant’s’ age.

### Data analysis

The psychophysical data analysis used binocular measurements. Inverse cumulative Gaussian fit curves were used to optimize the lateral masking parameters. The data were fitted to an error function of the equation

y=(U‐L)*(erf((x‐A)/B)+1)/2+L

where %A is the horizontal position of the point of inflection of the fitted function, %B controls the slope of the fitted function, %L is the lower asymptote of the fitted function, and %U is the upper asymptote of the fitted function. The best fitting parameters were obtained through least mean square fitting which minimizes the sum of the difference between the data and the desired fit (the error) using available MATLAB functions.

The SPSS 20 Production Facility was used to analyse the data. A two-tailed independent t-test was performed by comparing the contrast sensitivity and the OCT measurements of the young vs. teenage groups. The level of significance was set at p<0.05. Pearson's correlation gave the correlation between contrast sensitivity, central macular thickness (i.e., foveal thickness) and age and for contrast sensitivity with foveal thickness. All parameters were tested for normality using skewness test.

Only OCT data for one eye from each subject were analysed. If scans were available from both eyes, then only the measurements from the right eye were included. The OCT measurements were analysed using Macular Cube maps. The average foveal thickness and the total macular volume were automatically calculated by an algorithm and presented as numeric values. The parafoveal width was measured manually from the OCT scan.

## Results

Forty-seven subjects aged 3–17 years participated in the study (Table 1). Forty-three subjects participated in lateral masking task (22 subjects in the young group and 21 subjects in the teenage group). Twenty-two subjects participated in the OCT study (11 subjects in the young group and 11 subjects in the teenage group. All subjects had normal visual acuity (0.03 LogMar±0.03) with a range of 0–0.1 LogMar and normal stereo acuity (38.19±22.07) with a range of 20–100 seconds of arc.

**Table 1:** Distribution of subjects (1 year = 12 months).

Age (Year)	No. of subjects	Mean age	SD
3	7	3.39	0.31
4	7	4.41	0.31
5	6	5.37	0.28
6	5	6.32	0.26
7	3	7.33	0.42
8	3	8.2	0.26
9	3	9.23	0.25
10	2	10.25	0.35
11	2	11.25	0.35
12	2	12.10	0.14
13	1	13	0
14	1	14	0
15	1	15	0
16	2	16.45	0.21
17	2	17.45	0.49

As previously suggested [61] and similarly to our recent study [3], the young subjects (3–6 years) demonstrated a mean crowding effect of 0.5 ± 0.05 log units, while the teenage subjects (7–17 years) exhibited no crowding effect (0.001 ± 0.03 log units, t(45) = 9.06, p<0.0001).

**Fig 1:**
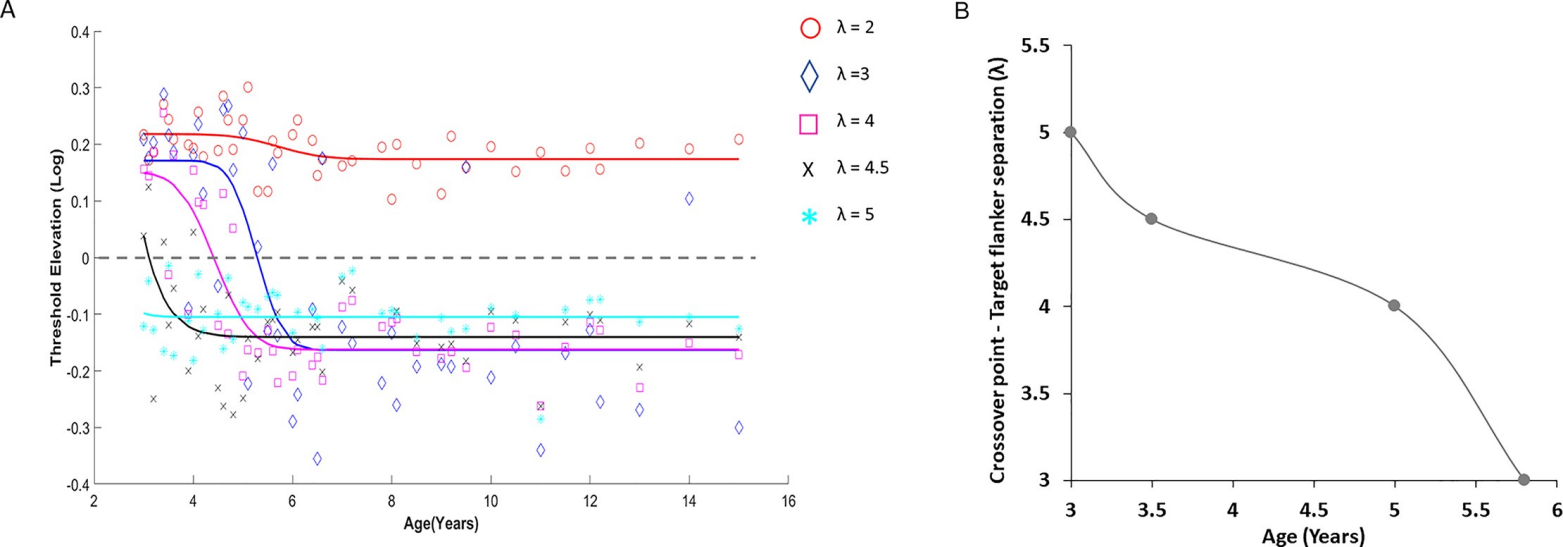
Effect of age on suppression and facilitation of various lateral target-flanker separations (2–5λ). **(A)** Threshold elevation at 9 cpd plotted against age (years, Y) for 2–5λ target-flanker separation. The lines denote inverse cumulative Gaussian fit curves (red circles—2λ, blue rhombus- 3λ, pink squares—4λ, black X– 4.5λ and turquoise asterisks—5λ). **(B)** The crossover point between suppression and facilitation calculated from Fig 1A as a point of y = 0 for each age line (indicating occurrence of facilitation) shown as target–flanker separation plotted against age.

Suppression was seen at a small target-flanker distance (2λ) in all the participants, but facilitation varied at larger target-flanker distances (3–5λ) depending on the participant’s age (Fig 1A). Children aged 3–5 exhibited a facilitation effect mainly at the 4–5λ target-flanker distance, while children above 6 years exhibited the adult pattern of collinear facilitation [24, 30, 31, 46, 62]. The relationship between age and threshold elevation (contrast detection of a single target in the presence of a mask normalized to the target condition) showed decreased suppression with increasing age, due to an increase in the facilitation effect at target-flanker separations larger than 3λ for all ages (r = 0.74, 3λ; r = 0.72, 4λ; r = 0.46, 4.5λ; r = 0.07, 5λ, p<0.05). We calculated the target-flanker separation at the crossover point (y = 0) where suppression turned to facilitation. The crossover differed with age (Fig 1B). At age 3, the crossover (i.e., facilitation) appeared at about 5λ, at 3.5 years it was about 4.5λ, at 5 years it was about 4λ, and at the age of 5.5–6 years it was about 3λ.

**Fig 2:**
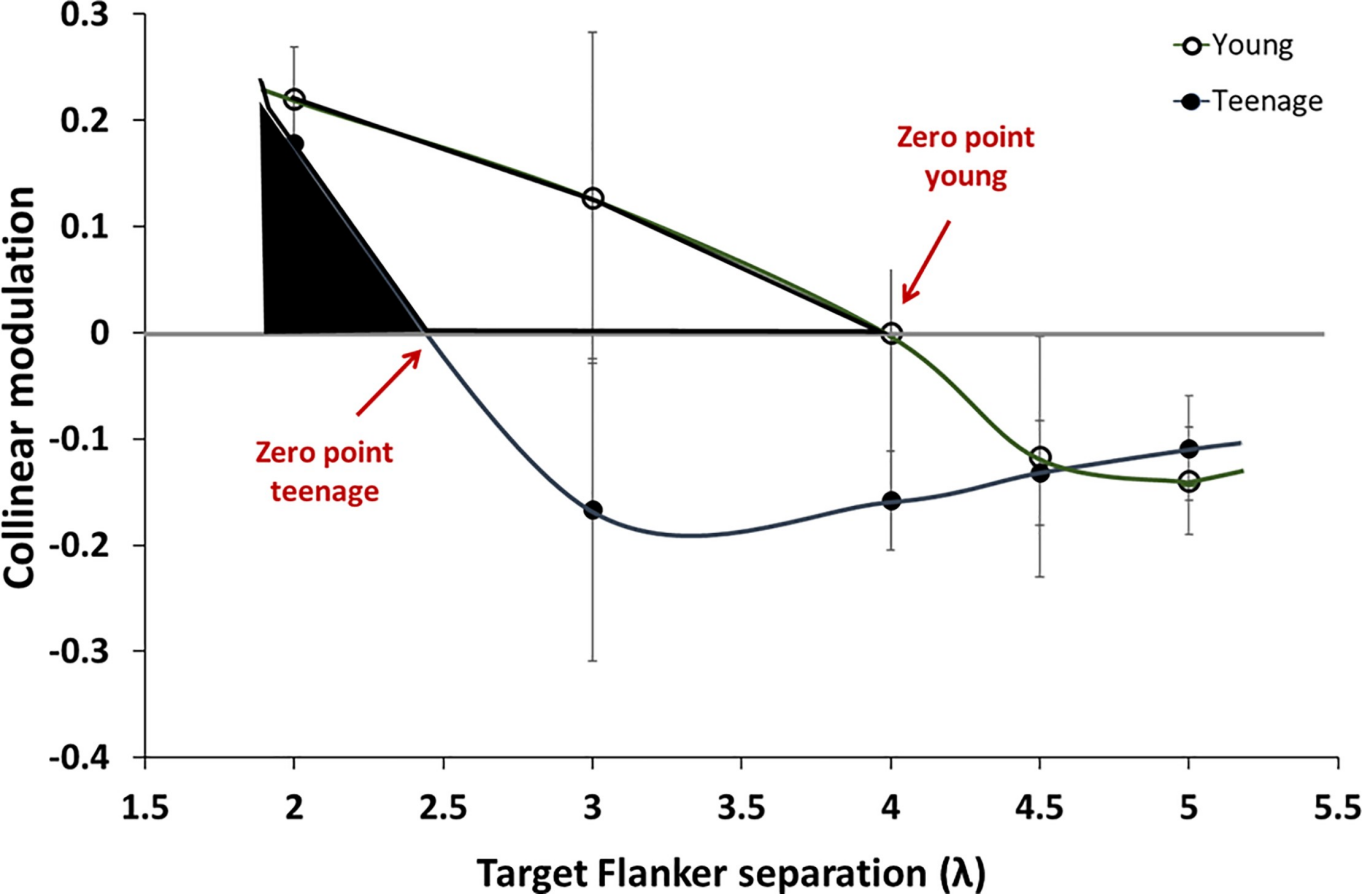
The suppression zone may reveal perceptive field size. An illustration of suppression and facilitation in young children and teenagers. The heavily enclosed areas show the difference between the suppression zones, i.e., the PF size for the younger (open) and the older children (filled). Error bars indicate the standard deviation of the mean.

Lev and Polat [33, 42] suggested that the distance at which the suppression turns to facilitation provides an estimate of the size of the PF. Fig 2 shows the average results of collinear modulation (patterns of collinear GPs presented within the context of other GPs) obtained from the lateral masking task. This figure demonstrates the estimation of PF size for the two age groups. We used the crossover point where collinear suppression was transformed to facilitation (y = 0) as the crossing border of the PF [33, 42]. The suppression zone in young children (3–6 years) was about 4λ, while in the teenage group it was about 2–2.5λ. The PF reached the adult size at an age of about 6 years (Fig 1).

**Fig 3:**
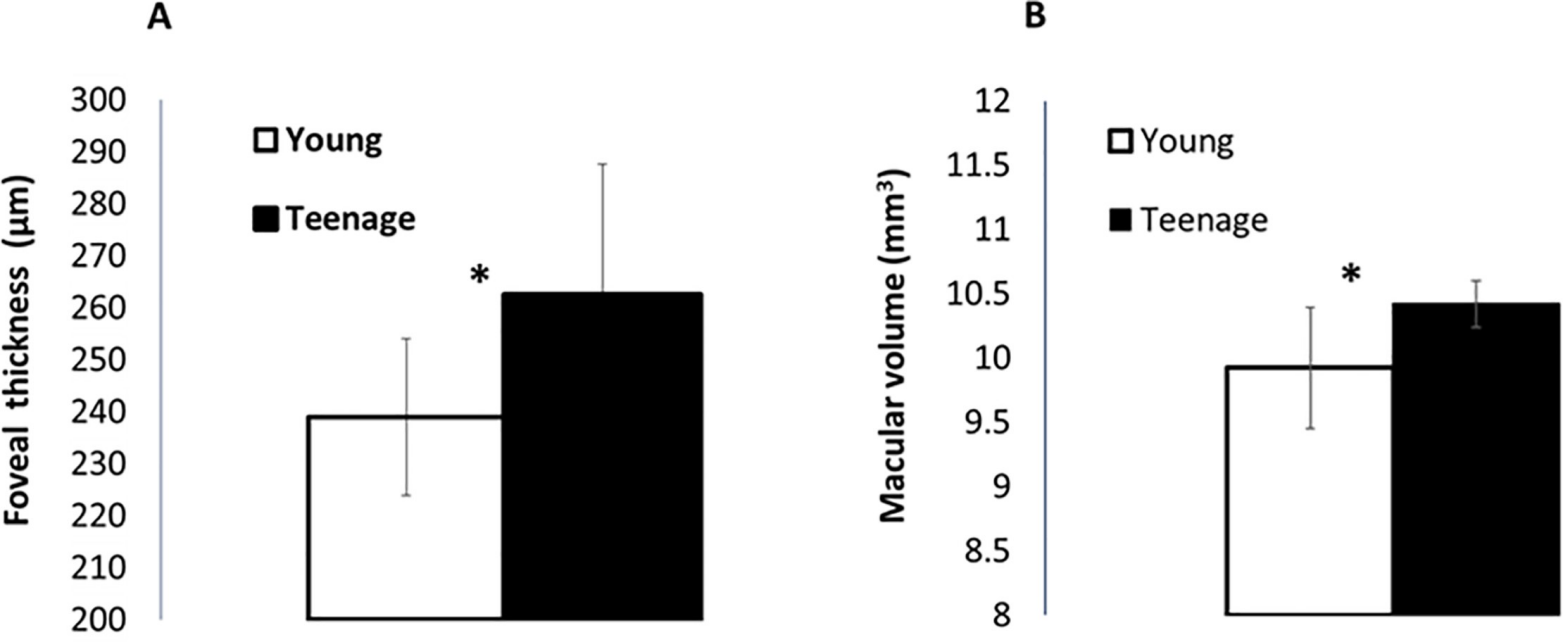
OCT measurements of foveal thickness (μm) and macular volume (mm^3^) in young and teenage participants. **(A)** Mean difference of the foveal thickness. **(B)** Mean difference of the macular volume. Bars indicate standard deviation of the mean.

Mean foveal thickness for the young group was lower (238.27 μm ± 15.79) than that for the teenage group (262.00 μm ± 19.72; Fig 3A). This differences was statistically significant (t(20) = -3.11, p = 0.005). Foveal thickness was normally distributed, with skewness of 0.5 (SE = 0.49). The mean macular volume in the young group (9.90 mm^3^ ± 0.18) was significantly lower than that in the teenage group (10.28 mm^3^ ± 0.48; t(20) = -2.44, p = 0.02; Fig 3B). Macular volume was normally distributed with skewness of 0.9 (SE = 0.49). There was no significant difference in the parafoveal width between the young group (2073.64 μm ± 142.33) and the teenager group (2121.27 μm ± 129.33, P = 0.42). Parafoveal width was normally distributed with skewness of -0.7 (SE = 0.49).

As previously suggested [14] and similarly to our recent study [3], mean contrast sensitivity in the young group (21.19 (100/ contrast threshold) ± 5.38) was significantly lower than that of the teenage group (34.94 (100/threshold contrast) ± 6.41; t(20) = 8.07, p = 0.000). Contrast sensitivity was normally distributed with skewness of 0.19 (SE = 0.49).

Strong correlation was found between foveal thickness and age (r(22) = 0.73, p = 0.000). Weak correlation was found for macular volume with age (r(22) = 0.57, p = 0.06). In addition, contrast sensitivity was strongly correlated with age (r(22) = 0.96, p = 0.000).

**Fig 4:**
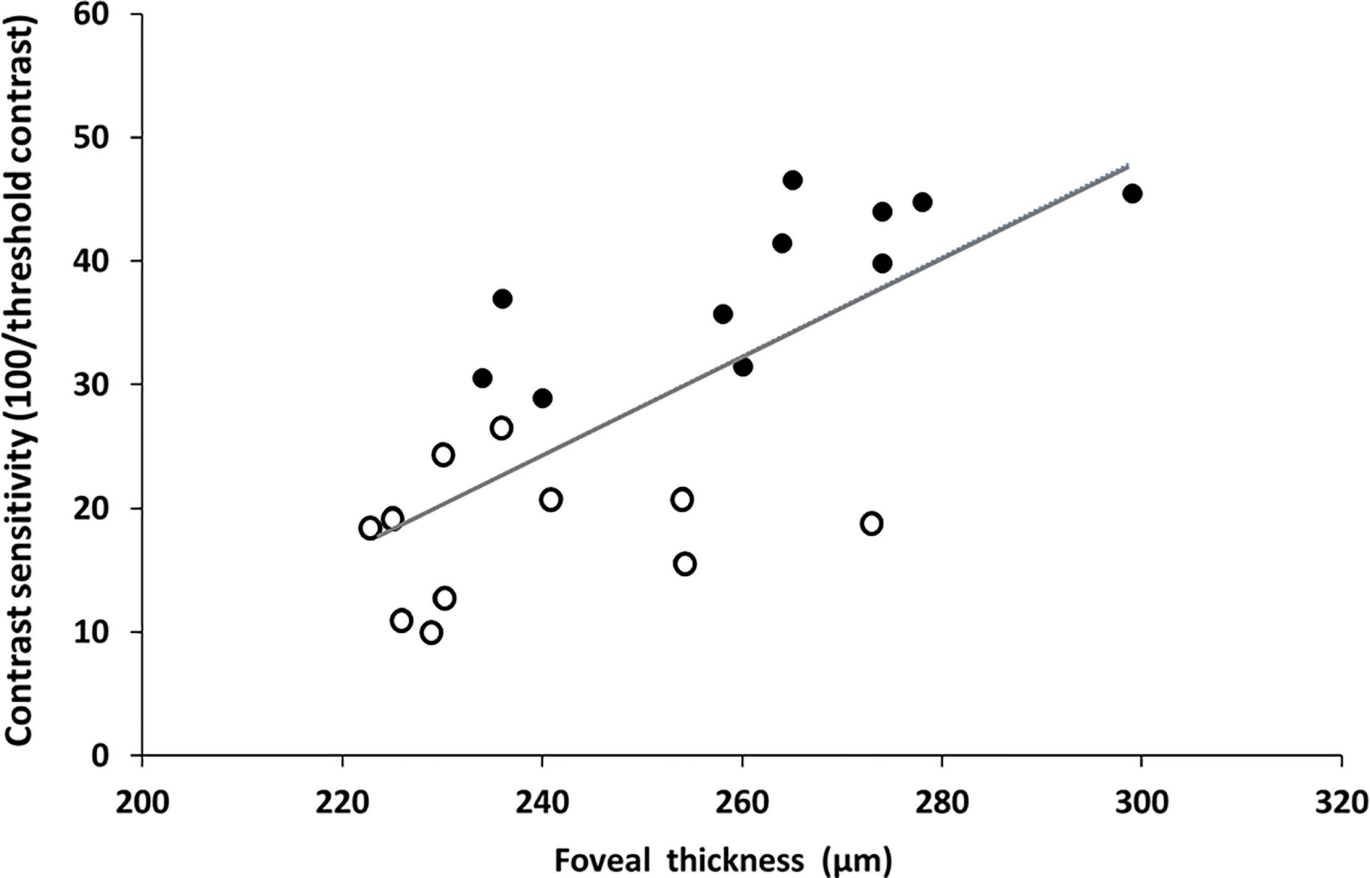
Foveal development vs. visual performance. Correlation between contrast sensitivity and foveal thickness. Open dots represent young children; filled dots represent the teenaged group.

We studied the link between foveal development and contrast sensitivity in both age groups. As shown in Fig 4, a significant correlation was found between foveal thickness and contrast sensitivity (r(22) = 0.71, p = 0.000).

## Discussion

Although it is well known that several visual functions, such as visual acuity, contrast sensitivity and contour detection, develop throughout childhood, little is known about how other functions, such as crowding and lateral interaction, affect the development of visual perception. In addition, there is a lack of information on the effects of the sequence of visual maturation (fovea and the primary visual cortex) on basic visual functions and perception. This study examines some of these aspects.

Doron et al. (2015) suggested a sequence of development of visual functions (i.e., contour integration, crowding effect and contrast sensitivity) throughout the maturation period from early childhood until an adult-like performance is achieved. They hypothesized that maturation of these visual processes can be explained by changes in neuronal mechanisms underlying the shift from suppression at a young age to facilitation in older children. This effect may be consistent with a close relationship between masking and crowding [42], suggesting that facilitation balances the crowding effect. That is, young children are more affected by the proximity of collinear flankers at 3λ (showing suppression rather than adult-like facilitation) and exhibit a strong crowding effect. With increasing age, the crowding effect decreases, reaching the adult level at about 6 years, which is the approximate age that facilitation appears. Development of the visual proximity effect was also reported in a study testing the effects of collinearity and spatial proximity on contour integration [63]. This study showed that the ability of young children to detect contour integration is limited by proximity regardless of collinearity.

The current study showed that young children exhibited a facilitation effect mainly at a 4–5λ target-flanker distance, while children above 6 years exhibited the adult pattern of lateral interactions [24, 30, 31, 46, 62]. That is, young children did present a facilitation effect, but it differed from that of adults. Our results also support the measure of the suppressive zone being indicative of the size of the PF [33, 42]. We thus speculate that the absence of facilitation at 3λ in young subjects is due to the size of the PF (suppression zone), which is still larger than that of the adult fovea and is similar to the PF size at the periphery. These results support a link between poor visual functions (i.e., suppression/crowding) and PF size [64]. Our study suggests that between the ages of about 3 and 6 the improvements in crowding (and contour integration tasks [3]) are caused by a gradual reduction in the size of the suppression zone with increasing age. From about age 6 onwards reduction of crowding is due to the development of facilitation. This process occurs in parallel with the development of contrast sensitivity [3].

In addition to the maturation process occurring at the cortical level and those suggested above, there are known developmental processes in the retina. To better understand the cascade of visual development this study examined changes in the fovea using the OCT method, as well as contrast sensitivity. Here we described changes in foveal thickness and macular volume in some of the same subjects who showed a perceptual development. As in previous studies [13, 65] our OCT results showed that teenagers (exhibiting values similar to adults) had a significantly thicker central fovea than young children, as well as a higher macular volume. Moreover, there was a significant correlation of both foveal thickness and macular volume with age. These results are consistent with previous findings [66]. Furthermore, combining the results of both age groups, (i.e., a general view of the development sequence) showed a link between contrast sensitivity and foveal thickness. Hence, since photoreceptor packing is considered to affect visual acuity [14–16], it is reasonable to suggest that age (i.e., epiphenomena) is not the only factor contributing to development of contrast sensitivity. Thus, these results support the idea that the maturation of contrast sensitivity is affected by foveal development (in addition to maturation of the visual cortex).

Our data do not distinguish which layers in the retina were responsible for the difference in foveal thickness between the age groups, but, given the centrifugal displacement of the inner retinal cells toward the rim of the fovea pit, the increasing thickness is most likely due to changes in the photoreceptor packing and increased number of axons [8, 13]. However, since the macula volume includes mean values from the fovea, parafovea and perifovea, the differences between age groups cannot be explained by only the foveal changes. Rather, quantitative and structural changes of a wider area around the fovea may have a major effect on foveal cone distribution [8].

The psychophysics and OCT measurements showed that most children who exhibited a high crowding level and masking (suppression) also exhibited a thinner central macula and lower contrast sensitivity. In contrast, most of the teenage group exhibiting no crowding and a facilitation effect had a thicker central macula and higher contrast sensitivity.

In addition, other physiological factors may contribute to perceptual maturation [67], such as the structural development of the perceptual visual networks up to late adolescence [18]. Intracortical myelin in the visual cortex increases up to adulthood [68, 69] and cortical thickness decreases with age [70, 71]. In addition, the ganglion cell layer in the fovea becomes thinner during development, being thinnest at around 6–16 years [13]. In contrast, the lateral geniculate nucleus reaches its adult morphology by the age of 9 months [72].

We are aware of the relatively small number of participants in the OCT study, which was due to parental objection to an OCT test. However, this number of participants was sufficient to give findings similar to those reported in previously, allowing us to derive information about the maturation process. Some of the participants were very young and could have had difficulty in focusing their attention. However, our study was designed to accommodate this possibility and the first author paid close attention when the young children performed the tasks. However, future studies may include eye movement tracking to rule out potential strategies in performing the tasks and to ensure maintained fixation on the stimulus.

This study used psychophysical and physiological approaches (imaging) in the same cohort to examine visual maturation processes. Our results suggest that foveal and perceptual changes may be part of a sequence of events in which retinal development effects contribute to maturation of contrast sensitivity, and the reduction in the perceptive field size may affect crowding. The quality of vision that develops throughout childhood is possible due to fovea development, i.e., older children and teenagers can detect optotype (or object) at a lower contrast than young children. A decrease in the crowding effect is made possible due to maturation of the perceptive field, i.e., older children and teenagers can identify an optotype (or object) when it appears in a line of other letters, contrasting with young children who can identify the same size of an optotype only if it is presented alone (uncrowded).

## Supporting information

S1 Fig OCT illustration. Line raster: The vertical line indicates the foveal thickness. The horizontal line indicates the parafoveal width.
(TIF)

S2 Fig Lateral masking stimuli. Target flanker separation (3λ): Flanker with target (left side) and flanker without target (right side).
(TIF)
