# Supplementary figures and images for: Development of global visual processing: From the retina to the perceptive field
Source: PLoS One. 2020 Aug 31;15(8):e0238246. doi: 10.1371/journal.pone.0238246 (PMC7458325; doi:10.1371/journal.pone.0238246)

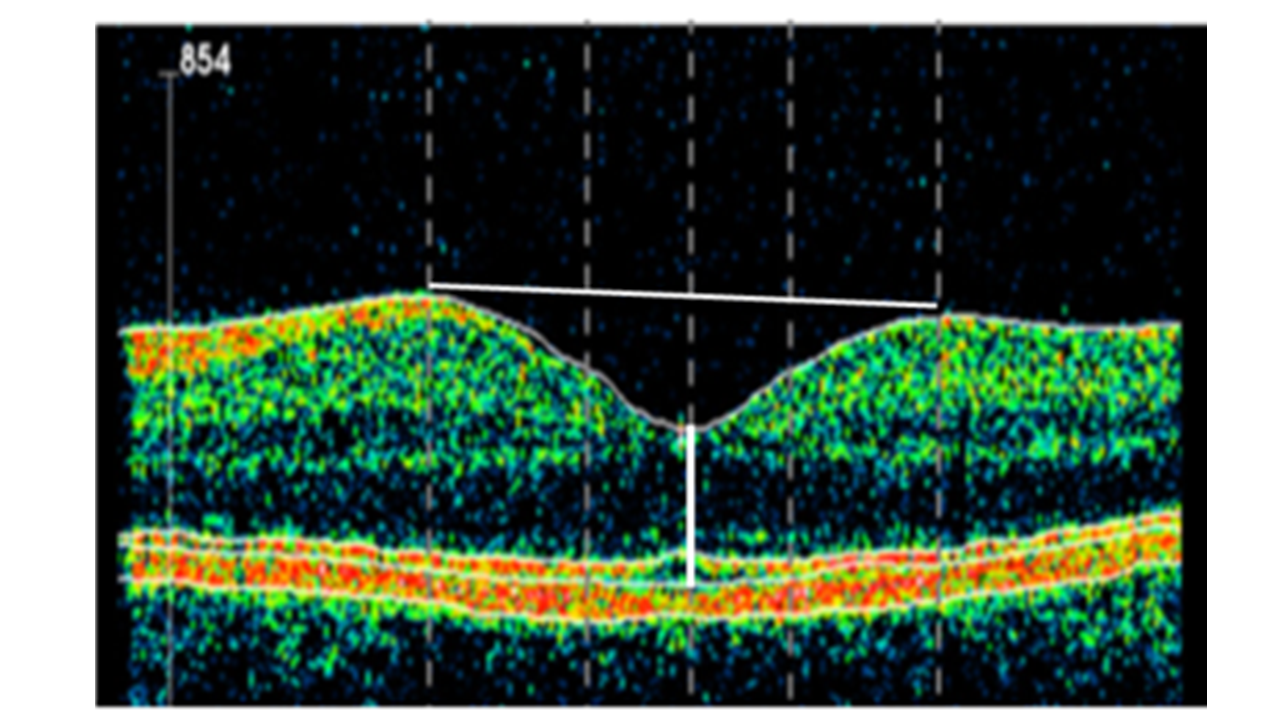

Supplement: S1 Fig — Line raster: The vertical line indicates the foveal thickness. The horizontal line indicates the parafoveal width. (TIF) [file pone.0238246.s001.tif]

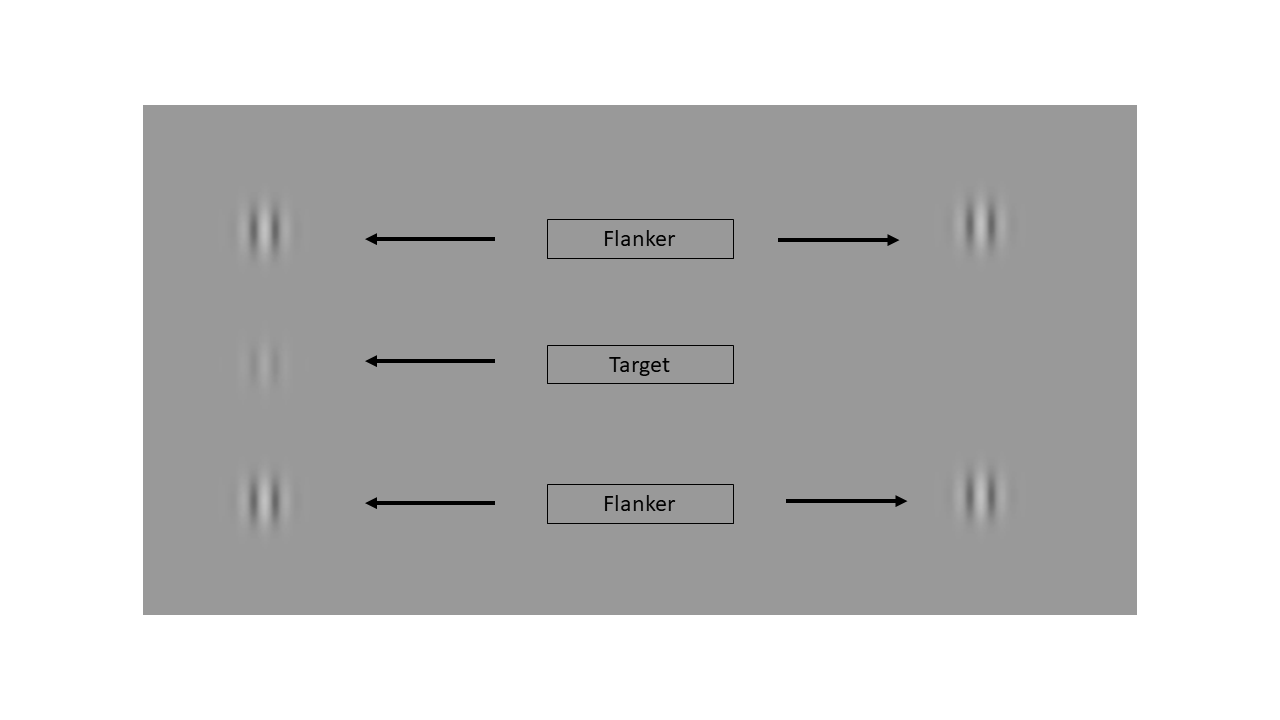

Supplement: S2 Fig — Target flanker separation (3λ): Flanker with target (left side) and flanker without target (right side). (TIF) [file pone.0238246.s002.tif]
